# Production of beneficial lignans in heterologous host plants

**DOI:** 10.3389/fpls.2022.1026664

**Published:** 2022-10-11

**Authors:** Tomotsugu Koyama, Jun Murata, Manabu Horikawa, Honoo Satake

**Affiliations:** Bioorganic Research Institute, Suntory Foundation for Life Sciences, Seikacho, Kyoto, Japan

**Keywords:** *Forsythia* species, heterologous production, lignans, podophyllotoxin, tobacco, sesamin

## Introduction

Plant specialized metabolites, such as alkaloids, flavonoids, terpenoids, and lignans, have health-promoting benefits for the world’s increasingly aging population. Lignans are phenylpropanoid dimers of various chemical structures with diverse health benefits (Suzuki and Umezawa, 2007; Rodríguez-García et al., 2019; Hano et al., 2021). Dietary lignans are obtained from oil seeds, cereal grains, legumes, vegetables, fruits, and beverage, and have attracted attention as food nutrients with potential health promoting activities (Durazzo et al., 2018; Rodríguez-García et al., 2019). Lignan-rich plants have been used in Chinese medicines (Teodor et al., 2020; Xu et al., 2022). (+)-Sesamin is a furofuran lignan, highly accumulated in *Sesamum indicum* (sesame) seeds, and commercially available as a health-promoting supplement. In mammals, (+)-sesamin metabolites attenuate oxidation and inflammation, thereby protecting the liver (Nakai et al., 2003; Kabe et al., 2020). (+)-Sesamin also exhibits several anti-tumor and anti-bacterial effects (Majdalawieh et al., 2017; Oikawa et al., 2022). (-)-Podophyllotoxin is an aryltetralin lignan found in the rhizomes of *Podophyllum* plants, and is utilized as a leading compound in anti-cancer drugs (Ardalani et al., 2017; Changxing et al., 2020).

Though (+)-sesamin and (-)-podophyllotoxin are important to human health, their plant sources are limited. Specific plant species accumulate large quantities of beneficial lignans, but model plants, such as *Arabidopsis thaliana*, produce them in small amounts (Umezawa, 2003a; Nakatsubo et al., 2008; Okazawa et al., 2011). Natural growth and agricultural cultivation of lignan-producing plants is frequently threatened owing to climate change. *S. indicum* plants, the strongest known synthesizers of (+)-sesamin, are constrained by environmental stress (Islam et al., 2016; Dossa et al., 2017; Dossa et al., 2019). *Podophyllum* species, natural sources of (-)-podophyllotoxin, grow naturally in very limited regions; hence they are an endangered species (Chaurasia and Tayade, 2012; Chaudhari et al., 2014; Singh et al., 2021). The regeneration of *Podophyllum* species is considerably slower than their harvest rate (Singh et al., 2021).

Cultured cells and hairy root lines from natural plant sources have been established *in vitro*, and developed to produce beneficial lignans (Fuss, 2003; Bayindir et al., 2008; Ragamustari et al., 2014; Lalaleo et al., 2018; Renouard et al., 2018; Bazaldúa et al., 2019; Mikac et al., 2021). Growth conditions to induce the accumulation of beneficial lignans have also been optimized practically (Satake et al., 2015; Renouard et al., 2018; Changxing et al., 2020). However, cost-effective scalable production of beneficial lignans using these culture systems is yet to be developed (Mikac et al., 2021).

Therefore, new plant sources are required to address these issues. The novelty of this article opinion is to highlight the prospects for the development of heterologous production of beneficial lignans such as (+)-sesamin and (-)-podophyllotoxin-related lignans, as transgenic cells and plants are excellent hosts for heterologous production of other beneficial chemicals (Lu et al., 2016; Arya et al., 2020; Wu et al., 2021). This is underpinned by the identification of the lignan-biosynthetic enzymes, and generation of transgenic plants expressing the lignan-metabolic enzyme genes as heterologous hosts.

## The heterologous hosts

### Heterologous production of (+)-sesamin and related lignans in *Forsythia* plants

**Figure 1:**
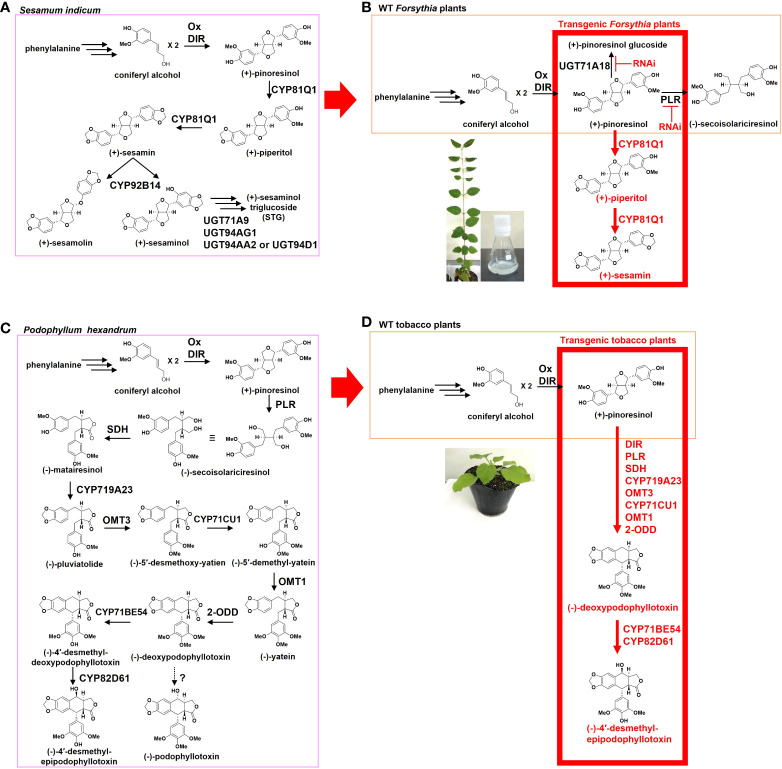
The heterologous production of beneficial lignans in transgenic plants. **(A)** *Sesamum indicum* lignan-metabolic pathway (pink). **(B)** Heterologous production of sesame lignans in *Forsythia* plants. Wild-type (WT) *Forsythia* plants biosynthesize (+)-pinoresinol (orange). Ectopic expression of *CYP81Q1* gene produces sesame lignans, (+)-piperitol, and (+)-sesamin in transgenic *Forsythia* plants (red). RNAi of PLR and UGT71A18 increase the accumulation of (+)-sesamin in transgenic *Forsythia* cells. **(C)** *Podopophyllum hexandrum* lignan-metabolic pathway (pink) **(D)** Heterologous production of *Podopophyllum* lignans in tobacco plants. WT tobacco plants biosynthesize (+)-pinoresinol (orange). Transient co-expression of DIR, PLR, SDH, CYP719A23, OMT3, CYP71CU1, OMT1, and 2-ODD genes produces (-)-deoxypodophyllotoxin in the leaves of tobacco (red). Co-expression of additional cytochrome P450 enzymes (CYP71BE54 and CYP82D61) produces (-)-4′-desmethyl-epipodophyllotoxin (etoposide precursor). DIR, Dirigent proteins; PLR, Pinoresinol-lariciresinol reductase; SDH, Secoisolariciresinol dehydrogenase; OM,: *O*-methyltransferase; 2-ODD, 2-oxoglutarate/Fe(II)-dependent dioxygenase.

The knowledge of lignan biosynthetic enzymes, from different plants, is necessary for the selection of hosts for heterologous lignan production. The biosynthetic pathways for the sesamin-related lignans have a common beginning in the conversion of phenylalanine to coniferyl alcohol (CA), followed by the dimerization of CA, by dirigent proteins (DIR), to form the lignan precursor (+)-pinoresinol (**Figure 1A**) (Davin et al., 1997; Davin and Lewis, 2000; Umezawa, 2003b).

*S. indicum* seeds accumulate (+)-sesamin, (+)-sesamolin, and (+)-sesaminol triglucoside (Beroza and Kinman, 1955; Bedigian, 2010). (+)-Pinoresinol is oxygenated and converted to (+)-sesamin by the piperitol/sesamin synthase enzyme, also known as CYP81Q1 (Ono et al., 2006) (**Figure 1A**). (+)-Sesamin is further oxygenated to either (+)-sesamolin or (+)-sesaminol by CYP92B14, depending on the position of the oxygenated carbon in the aromatic ring system of (+)-sesamin (Murata et al., 2017). (+)-Sesaminol is further glucosylated by uridine diphosphate (UDP)-glucosyltransferases and is accumulated as (+)-sesaminol triglucoside (Noguchi et al., 2008; Ono et al., 2020).

Heterologous production of sesame lignans has been achieved in *Forsythia*-based transgenic systems. *Forsythia* plants have characteristics suitable for the heterologous production of lignans. They are perennial shrubs, propagated vegetatively, and distributed widely (Rosati et al., 2007). They accumulate high levels of the lignan precursor (+)-pinoresinol, and other beneficial lignans (Umezawa, 2003b; Guo et al., 2007; Suzuki and Umezawa, 2007; Chang et al., 2008; Morimoto and Satake, 2013; Wang et al., 2018). Extracts of *Forsythia* plants have been used empirically in traditional medicines (Dong et al., 2017; Wang et al., 2018). The regulation of lignan biosynthesis has been extensively analyzed (Shiraishi et al., 2016; Sun et al., 2018).

*Forsythia* plants synthesize (+)-pinoresinol but not (+)-sesamin. Therefore, (+)-sesamin was expressed heterologously in transgenic *Forsythia* cells and plants (**Figure 1B**). Kim et al. (Kim et al., 2009) demonstrated the production of (+)-sesamin in double-transgenic *Forsythia koreana (Fk)* cells, CPi-Fk, which had been stably transformed with CYP81Q1 and pinoresinol lariciresinol reductase-RNA interference (PLR-RNAi) (**Figure 1B**). Since CYP81Q1 converts only (+)-pinoresinol aglycone into (+)-sesamin, and 90% of the (+)-pinoresinol is glucosylated in *Forsythia* wild type cells or leaves (Ono et al., 2006; Morimoto and Satake, 2013, Satake et al., 2015; Satake et al., 2016), the triple-transgenic *Fk* cells, U18i-CPi-Fk, were generated by stable transformation of CPi-Fk with an RNAi sequence against the (+)-pinoresinol-glucosylating enzyme, UGT71A18 (Ono et al., 2010). These U18i-CPi-Fk cells, in comparison with the CPi-Fk cells, showed approximately 5- and 1.4-fold increase in the synthesis of (+)-pinoresinol aglycone and (+)-sesamin, respectively (Murata et al., 2015). (+)-Sesamin production in U18i-CPi-Fk was upregulated approximately 3-fold, specifically under red light, for two weeks (Murata et al., 2015; Satake et al., 2016). These findings confirmed the potential of the *Fk*-transgenic cells for metabolic engineering-based lignan production, and paved the way for generating (+)-sesamin-producing *Forsythia* plants.

An illustration of the heterologous production of lignans in *Forsythia* plants: the generation of the transgenic *Forsythia* plants expressing sesame *CYP81Q1* gene results in the production of (+)-sesamin and its intermediate (+)-piperitol (**Figure 1B**) (Koyama et al., 2022). These transgenic *Forsythia* plants produced (+)-sesamin and (+)-piperitol sustainably, even after repeated vegetative propagation *via* explants (Koyama et al., 2022). Therefore, the transgenic *Forsythia* plants serve as prototype heterologous host plants, with the potential for mass propagation as alternative sources of (+)-sesamin and (+)-piperitol.

### Heterologous production of (-)-podophyllotoxin-related lignans in tobacco plants

In (-)-podophyllotoxin biosynthesis in *Podophyllum* plants, (-)-matairesinol, a downstream lignan of (+)-pinoresinol, is initially converted to (-)-pluviatolide by CYP719A23 (**Figure 1C**) (Marques et al., 2013). Methylation of (-)-pluviatolide is catalyzed by (-)-pluviatolide-*O*-methyltransferase (OMT3), which yields (-)-5′-desmethoxy-yatien. (-)-5′-Desmethoxy-yatien is hydroxylated to (-)-5′-demethyl-yatein by CYP71CU1 (Law and Sattely, 2015), which is further methylated by (-)-5′-demethyl-yatein-*O*-methyltransferase (OMT1) and converted to (-)-yatein. Thereafter, (-)-yatein is catalyzed by (-)-2-oxoglutarate/Fe(II)-dependent dioxgenase (2-ODD) to (-)-deoxypodophyllotoxin, the proposed precursor of (-)-podophyllotoxin. To date, the final step from (-)-deoxypodophyllotoxin to (-)-podophyllotoxin has not been identified (**Figure 1C**) (Law and Sattely, 2015). Instead, (-)-deoxypodophyllotoxin has been shown to be converted into (-)-4′-desmethyl-deoxypodophyllotoxin *via* demethylation by CYP71BE54, and is further metabolized by CYP82D61 to (-)-4′-desmethyl-epipodophyllotoxin, the direct precursor of etoposide (Law and Sattely, 2015).

(-)-Podophyllotoxin-related lignans were produced heterologously in *Nicotiana benthamiana* (**Figure 1D**). The cultivation of *N. benthamiana* has been established well by many research institutions and biotechnology companies, worldwide (Bally et al., 2018). Leaves of *N. benthamiana* are excellent hosts for the *Agrobacterium*-mediated transient gene expression assay, which has been developed well for high production of proteins and other compounds (Bally et al., 2018; Molina-Hidalgo et al., 2021). Thus, *N. benthamiana* is a promising heterologous host for the production of beneficial lignans.

The transient co-expression of *Podophyllum* (-)-podophyllotoxin-biosynthetic enzyme genes for CYP719A23, OMT3, CYP71CU1, OMT1, and 2-ODD, as well as previously known lignan-biosynthetic enzyme genes, metabolize exogenously applied (+)-pinoresinol to (-)-deoxypodophyllotoxin in the leaves of *N. benthamiana* (**Figure 1D**) (Law and Sattely, 2015). Furthermore, the transient co-expression of two additional cytochrome P450 enzymes, CYP71BE54 and CYP82D61, with the above-mentioned (-)-podophyllotoxin-biosynthetic enzymes, produced (-)-4′-desmethylepipodophyllotoxin (**Figure 1D**) (Law and Sattely, 2015). This study demonstrates the heterologous production of (-)-podophyllotoxin-related lignans in *N. benthamiana*.

The lignan precursor (+)-pinoresinol is biosynthesized *via* CA, and CA is produced from phenylalanine (CA pathway, **Figure 1D**), so to increase the supply of CA in the leaves of *N. benthamiana* transient co-expression of CA pathway enzymes was carried out (Schultz et al., 2019). Co-expression of 16 genes of the CA pathway, along with the (-)-podophyllotoxin-biosynthetic enzymes, greatly increased the content of (-)-deoxypodophyllotoxin in the leaves of *N. benthamiana* (Schultz et al., 2019). This co-expression system did not require exogenous application of (+)-pinoresinol, and thus, enabled *de novo* production of (-)-podophyllotoxin-related lignans in *N. benthamiana* leaves (Schultz et al., 2019). This study demonstrates the applicability of engineering to supply endogenous precursors to increase the yield of (-)-podophyllotoxin-related lignans in the leaves of *N. benthamiana.*

### Challenges for the heterologous production of beneficial lignans in microbes

Microbes are also important hosts for the heterologous production of plant specialized metabolites (Sato and Kumagai, 2013; Kotopka et al., 2018; Pyne et al., 2019; Birchfield and McIntosh, 2020). Previous studies have suggested the potential capability of bacteria to produce lignans. Two sequential *Escherichia coli* lines, harboring individual metabolic modules of (-)-matairesinol to (-)-5′desmetyl-yatein, and (−)-5′desmetyl-yatein to (−)-deoxypodophyllotoxin, reportedly metabolized exogenously applied (-)-matairesinol to (-)-deoxypodophyllotoxin (Decembrino et al., 2021). The human gut microbiome also metabolizes exogenously applied (+)-pinoresinol to various lignans (Xie et al., 2003; Espín et al., 2017; Bess et al., 2020). However, these bacteria require the exogenous application of lignan precursors to produce the beneficial lignans. The lignan precursor (+)-pinoresinol is, apparently, not produced by many bacteria. Therefore, it would be impossible to produce beneficial lignans without exogenous application of precursor lignans. Therein, *de novo* production of lignans would require synthetic biotechnology approaches that include tremendous bioinformatics and large-scale screening processes (Choi et al., 2019; Birchfield and McIntosh, 2020). In addition, ethical issues may arise in using the gut bacterium for the scalable production of lignans (Choi et al., 2019). Thus, the microbial production of beneficial lignans is more challenging than the plant-based production process.

## Discussion

The novelty of this article is to underscore the importance of lignan production in heterologous host plants. Transgenic expression of the lignan-biosynthetic enzyme genes in heterologous hosts, *Forsythia* and tobacco, is particularly promising for the production of (+)-sesamin and (-)-podophyllotoxin-related lignans, respectively. These heterologous lignan production strategies may circumvent the need for sesame and *Podophyllum* plants as natural sources of (+)-sesamin and (-)-podophyllotoxin-related lignans in future.

To develop more efficient transgenic plant-based lignan production systems, we emphasize the importance of increasing the lignan content in future studies. Introducing additional genes to activate the enzymes or stimulate the accumulation of the precursor (+)-pinoresinol in transgenic plants will pave the way for increasing the content of beneficial lignans. To activate cytochrome P450 enzymes that function in complex with native reductases (Murata et al., 2017), co-expression of cytochrome P450 enzymes with their native reductases may lead to an increase in the content of (+)-sesamin and (-)-podophyllotoxin-related lignans in transgenic *Forsythia* and tobacco plants, respectively. Introducing RNAi into the lignan-metabolic pathway stimulates the accumulation of (+)-pinoresinol in transgenic *Forsythia* cell lines (**Figure 1B**) (Kim et al., 2009b; Murata et al., 2015), therefore, co-expression of (-)-podophyllotoxin-metabolic enzymes with an RNAi construct to stimulate the accumulation of (+)-pinoresinol may increase the content of (-)-podophyllotoxin-related lignans in *N. benthamiana*. Similarly, activation of the CA pathway stimulates the accumulation of (+)-pinoresinol in *N. benthamiana* (**Figure 1D**) (Schultz et al., 2019); thus, co-expression of CYP81Q1 with the CA pathway enzymes may increase the content of (+)-sesamin in transgenic *Forsythia* plants. Furthermore, since the elicitor treatments and light changes induce the production of beneficial lignans in cultured cells and hairy root lines (Satake et al., 2015; Changxing et al., 2020; Markulin et al., 2021; Mikac et al., 2021), it is important to optimize the growth conditions also of the heterologous host plants in future studies. Finally, other lignan-rich plants, such as flax species (Markulin et al., 2021), are expected to be useful heterologous hosts for the transgenic expression of lignan biosynthetic enzyme genes.

## Author contributions

TK, JM, and HS wrote the manuscript, and TK and MH prepared the figure. All the authors approved the submitted version.

## Conflict of interest

The authors declare that the research was conducted in the absence of any commercial or financial relationships that could be construed as a potential conflict of interest.

## Publisher’s note

All claims expressed in this article are solely those of the authors and do not necessarily represent those of their affiliated organizations, or those of the publisher, the editors and the reviewers. Any product that may be evaluated in this article, or claim that may be made by its manufacturer, is not guaranteed or endorsed by the publisher.
